# Mineral Phases and Release Behaviors of As in the Process of Sintering Residues Containing As at High Temperature

**DOI:** 10.1155/2014/260504

**Published:** 2014-03-02

**Authors:** Xingrun Wang, Fengsong Zhang, Zexi Nong

**Affiliations:** ^1^State Key Laboratory of Environmental Criteria and Risk Assessment, Chinese Research Academy of Environmental Sciences, Beijing 100012, China; ^2^School of Chemical & Environmental Engineering, China University of Mining & Technology (Beijing), Beijing 100083, China

## Abstract

To investigate the effect of sintering temperature and sintering time on arsenic volatility and arsenic leaching in the sinter, we carried out experimental works and studied the structural changes of mineral phases and microstructure observation of the sinter at different sintering temperatures. Raw materials were shaped under the pressure of 10 MPa and sintered at 1000~1350°C for 45 min with air flow rate of 2000 mL/min. The results showed that different sintering temperatures and different sintering times had little impact on the volatilization of arsenic, and the arsenic fixed rate remained above 90%; however, both factors greatly influenced the leaching concentration of arsenic. Considering the product's environmental safety, the best sintering temperature was 1200°C and the best sintering time was 45 min. When sintering temperature was lower than 1000°C, FeAsS was oxidized into calcium, aluminum, and iron arsenide, mainly Ca_3_(AsO_4_)_2_ and AlAsO_4_, and the arsenic leaching was high. When it increased to 1200°C, arsenic was surrounded by a glass matrix and became chemically bonded inside the matrix, which lead to significantly lower arsenic leaching.

## 1. Introduction

Arsenic pollution in solid wastes has aroused significant environmental concerns. Arsenic is toxic and it may result in skin lesions and cancer of the liver, kidneys, and stomach [1, 2]. Besides, it is hard to dispose arsenic. Arsenic is widely used in industrial plants and many ores are associated with arsenic, such as iron, gold, silver, mercury, copper, lead, and zinc minerals [3]. In China, about 30,000 t of arsenic enters into nonferrous metal smelting plant every year. arsenic residues are mainly discharged into waters and simply stacked in piles, which has caused severe contamination in some areas, for example, Dasha river arsenic contamination in Henan province and Yangzonghai arsenic contamination in Yunnan province. Therefore, it is urgent to safely, stably, and harmlessly transform arsenic-containing wastes into resources.

In solid waste and contaminated soil, arsenic exists mostly as As(III) and As(V). As(III) is generally regarded as being more mobile and toxic than As(V) [4, 5], while the latter is more easily adsorbed onto the surface of wastes. X-ray absorption near edge structure (XANES) spectroscopy has been increasingly used to determine the oxidation states in arsenic-containing wastes. Extended X-ray absorption fine structure (EXAFS) spectroscopy has been used to investigate the As contamination status of local environment [6–8].

Stabilization/solidification (S/S) processes are widely used to immobilize As in solid wastes [11]. Numerous researchers have studied the S/S process to immobilize As by means of various S/S agents such as cement [12–14], fly ash [15–17], and cement kiln dust [18, 19]. It has been reported that the Ca-As precipitates were the major As immobilization mechanisms [20, 21]. Specific Ca-As precipitates, such as Ca_3_(AsO_4_)_2_, CaHAsO_3_ [20, 22], Ca–As–O, Ca_4_(OH)_2_(AsO_4_)_2_·4H_2_O, and NaCaAsO_4_·7.5H_2_O [16, 19], have been reported as the compounds responsible for As immobilization in S/S processes. The amount of solid waste is increased greatly after S/S processes due to the adding of S/S agents and the use of wastes after S/S processes is also an urgent problem waiting for solution.

Ceramic, as a kind of lightweight aggregate, is often used to produce concrete mixtures. It is common to use clay producing ceramic nowadays [23], but it requires too much natural resources. The total lightweight aggregate output in China in 2004 is about 420 million m^3^. Therefore, the use of arsenic-containing wastes as a possible substitute of clay has the potential to provide plenty of raw materials while reducing the consumption of clay. At the same time, the environmental impact caused by arsenic-containing wastes can also be reduced. Earlier studies on making lightweight aggregate from wastes focused mainly on using sewage sludge [24–26] and fly ash [27, 28] as an additive material to clay, but few are particularly devoted to the sintering of arsenic-containing wastes and related mechanisms. During sintering, many reactions and transformations will happen in phyllosilicates and the accompanying minerals, like quartz, calcite, feldspar, hematite, and dolomite, which result in the formation of various crystalline phases. All these transformations and products play an important role in volatilization release and leaching of arsenic.

The aim of this work is to study the volatilization release and leaching of arsenic under different sintering conditions, including sintering temperature and sintering time, such as arsenic volatilization rate, as well as the arsenic leaching behavior of the sintered products. Besides, the reaction mechanism of As in sintering is analyzed by mineralogy and the microstructure of sintered products.

## 2. Materials and Methods

### 2.1. Sintering Lightweight Aggregates

As-containing residues in this study were obtained from a chemical plant in Henan province, which produced sulfuric acid through sintering pyrite containing As at high temperature, and other materials, such as clay and coal ash, were collected from a cement plant in Beijing (as shown in Table 1). The size distribution of residues was as follows: 41.71% were 20 mesh < *x* < 100 mesh and 46.19% were *x* > 100 mesh.

As-containing residues, clay, and coal ash were mixed together. Cylindrical specimens with a diameter of 10 mm and a weight of 2 g were prepared by uniaxial pressing of 10 MPa and subsequently sintered in an electric kiln with sintering temperatures ranging from 1000 to 1350°C for 15, 30, 45, and 60 min, respectively. The specimens were taken out and cooled at room temperature. A ramp rate of 9°C/min was used.

### 2.2. Characterization

Heavy metals in residues and in sintered products were determined according to an ASTM method after the samples were digested [29]. The elements were measured by inductively coupled plasma mass spectrometry (ICP-MS, 7500Cs, Agilent Technologies) [30].

Fixed rate of As was defined as the ratio of As weight in products after sintering to that before.

The leaching toxicity of heavy metals in residues and sintered products was analyzed with horizontal vibration extraction procedure (HVEP) [31] and toxicity characteristic leaching procedure (TCLP) [32], respectively. HVEP adopted deionized water as extractant and TCLP used 1# extractant, that is, mixed CH_3_COOH/NaOH solution with pH = 4.93. The leaching solution was filtrated with 0.45 um millipore filter and tested by inductively coupled plasma mass spectrometry (ICP-MS, 7500Cs, Agilent Technologies).

Si, Ca, Mg, Fe, Al, and K contents were determined using an X-ray fluorescence spectrometer (XRF, Shimadzu Lab Center XRF-1700, Japan).

X-ray diffraction (XRD, Seimens O8DISCOVER) using 40 mA and 40 kV Cu K_radiation was used for the research of crystalline phases in sintered products. The crystalline phases were identified by comparing the intensities and the positions of the Bragg peaks with the data files of the Joint Committee on Powder Diffraction Standards (JCPDS).

The surface characteristics of sintered products were analyzed visually with scanning electron microscope (SEM). In this study, it adopted KYKY2000 SEM produced by KYKY Technology Development Ltd.; the resolution ratio of secondary electron image point was 6 nm and the magnification factor was 15~100000 times.

### 2.3. Experimental Devices

**Figure 1:**
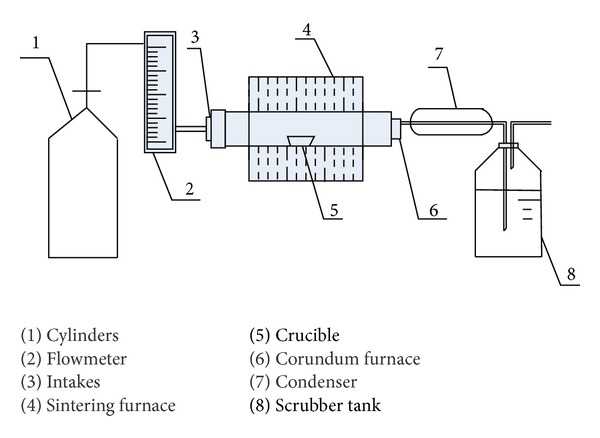
The structure of sintering device.

The sintering device was shown in Figure 1. Place the specimen in corundum crucible and then push it into high temperature corundum furnace for sintering.

## 3. Results and Discussion 

### 3.1. Properties of As-Containing Residues

Table 1 shows the chemical composition of As-containing residues, clay, and coal ash determined by XRF. The residues contain large amounts of Fe_2_O_3_, with concentrations of 69.78%. The secondary components are SiO_2_ (10.10%), Al_2_O_3_ (3.16%), and CaO (4.97%). The contents of other oxides are all less than 2%. To get fine lightweight aggregate, the chemical composition of raw material used should be similar to clay and satisfy the following requirements: SiO_2_ (48–70%); Al_2_O_3_ (8–25%); Fe_2_O_3_ + FeO (3–12%); CaO + MgO (1–12%) [33]. The compositions of residues are not suitable for sintered lightweight aggregate. According to the composition of Ca, Si, Al, and Fe as required by sintering lightweight aggregates, As-containing residues, clay, and coal ash were mixed together, and the mass ratio was 1 : 16 : 3, and following studies are based on the above mass ratio.

Table 2 shows the trace elements in the residues samples determined by ICP-MS. The major heavy metal identified is As, with concentrations of 5552.40 mg/kg. The secondary components are Zn (1279.24 mg/kg) and Ba (1046.80 mg/kg), while the other heavy metals are all less than 300 mg/kg. The second line of Table 2 gives the HVEP results of heavy metals, and as it indicated, the leaching concentration of As (8.35 mg/L) does not only exceed China Environmental Quality Standards for surface water [10] (0.1 mg/L) but also is higher than China Identification Standard for hazardous wastes [9] (5 mg/L). The leaching concentrations of other heavy metals are all in the range of China Identification Standard for hazardous wastes and China Environmental Quality Standards for surface water.

### 3.2. Sintered Products at Different Sintering Temperatures

#### 3.2.1. Volatilization of As at Different Sintering Temperatures

**Figure 2:**
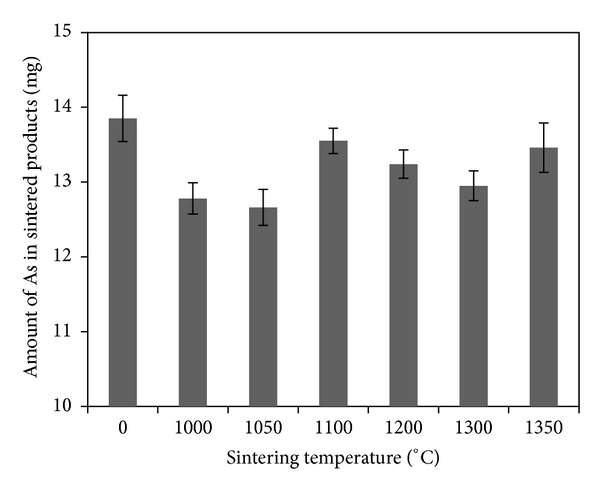
Effect of sintering temperature on the total amount of As in sintered products.

**Figure 3:**
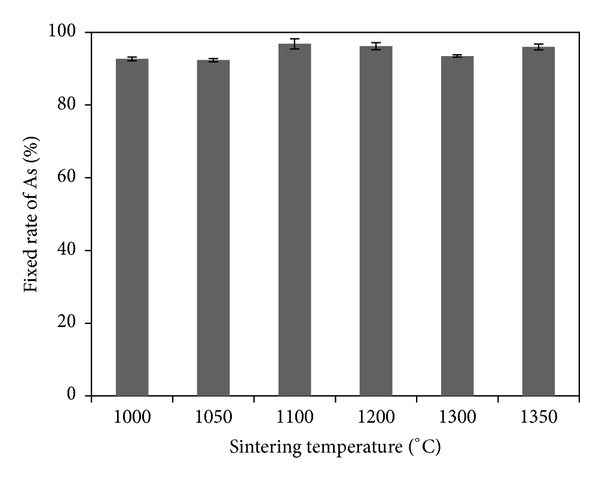
The fixed rates of As in sintered products at different sintering temperatures.

Figures 2 and 3 show the total amount of As in sintered products and the fixed rate of As (the specimen was not sintered at 0°C). As indicated in Figure 2, after sintering at different temperatures, the total amount of As was reduced (12.64~13.46 mg) compared with that before sintering (13.65 mg), indicating that arsenic was volatilized during sintering.  Figure 3 shows that the fixed rates of As in sintered products were high (92.58%~98.62%) at different sintering temperatures. Thus, the volatilization rate of As was low during sintering and above 90% As was fixed in sintered products.  Figure 3 also reveals that the volatilization rate of As does not have significant relationship with sintering temperatures between 1000 and 1350°C.

#### 3.2.2. As Leaching Behavior of Sintered Products at Different Sintering Temperatures

Leaching toxicity test is the most common method to test the characteristics of heavy metal released into the environment. In this study, HVEP and TCLP were used to survey the characteristics of As leaching of the sintered products.

Table 3 lists the As leaching concentration of sintered products at different sintering temperatures. It indicated that with HVEP, as the sintering temperature increased to 1000°C, the leaching concentration was enhanced significantly, from 0.568 mg/L to 0.962 mg/L, indicating that arsenic mineral phase in sintered products was changed and As in the sintered products was more easily released into the environment. When the temperature continued to rise to 1200°C, the As leaching concentration decreased significantly to 0.002 mg/L. With TCLP, a similar phenomenon was shown. From room temperature to 1000°C, As leaching concentration in sintered products increased from 0.751 mg/L to 1.444 mg/L, and as the temperature raised to 1200°C, As leaching concentration decreased significantly to 0.0017 mg/L. As the temperature continued to rise, the concentration changed a little. It is concluded that in order to reach lower leaching concentration of As, the sintering temperature must be higher than 1200°C. So, the following studies are based on the sintering temperature of 1200°C.

### 3.3. Sintered Products at Different Sintering Times

#### 3.3.1. Volatilization of As at Different Sintering Times

**Figure 4:**
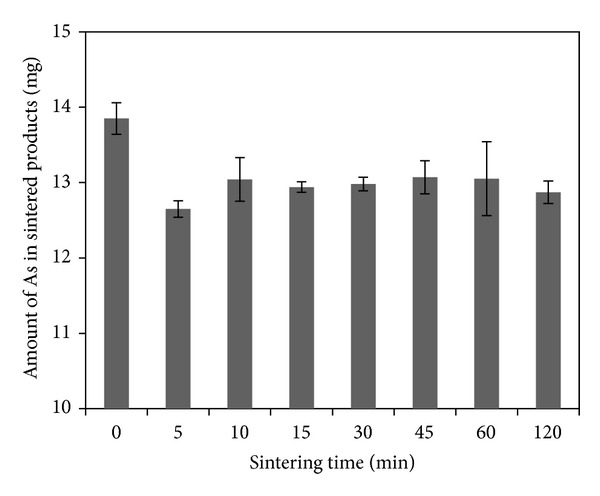
Effect of sintering time on the total amount of As in sintered products.

**Figure 5:**
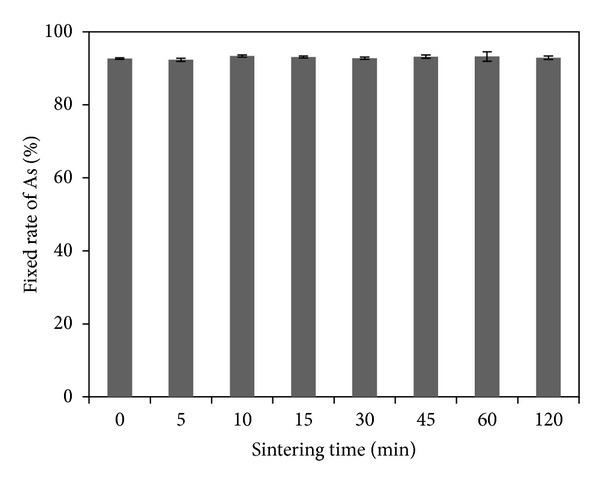
The fixed rates of As in sintered products at different sintering times.

With 2000 mL/min air inflow and 1200°C sintering temperature, specimen was sintered for different times. As shown by Figure 4 (the specimen was not sintered at 0 min), the total amount of As was lower after sintering than before.  Figure 5 shows the fixed rates of As in sintered products for different sintering times. As indicated by the figure, after 5 min sintering, the fixed rate of As was up to 92.53%, and as the time extended, the rates did not change correspondingly. More than 90% of As remained in sintered products. It is concluded that arsenic volatilization occurred within 5 minutes and after 5 min sintering arsenic volatilization changed a little.

#### 3.3.2. As Leaching Behavior of Sintered Products at Different Sintering Times

Table 4 displays the leaching concentration of As at different sintering times at the conditions of 10 MPa molding pressure, 2000 mL/min air inflow, and 1200°C sintering temperature. As indicated by the table, with HVEP, the longer the sintering time, the lower the As leaching concentration. At 45 min, the concentration was reduced to 0.003 mg/L. With TCLP, at 30 min, the concentration reduced to 0.0023 mg/L and as the time extended >30 min, the concentration changed a little. It is concluded that the sintering time must be no less than 45 min for the lower leaching concentration of As. So, some studies are based on the condition of 45 min in the following text.

### 3.4. Structural Characterization

**Figure 6:**
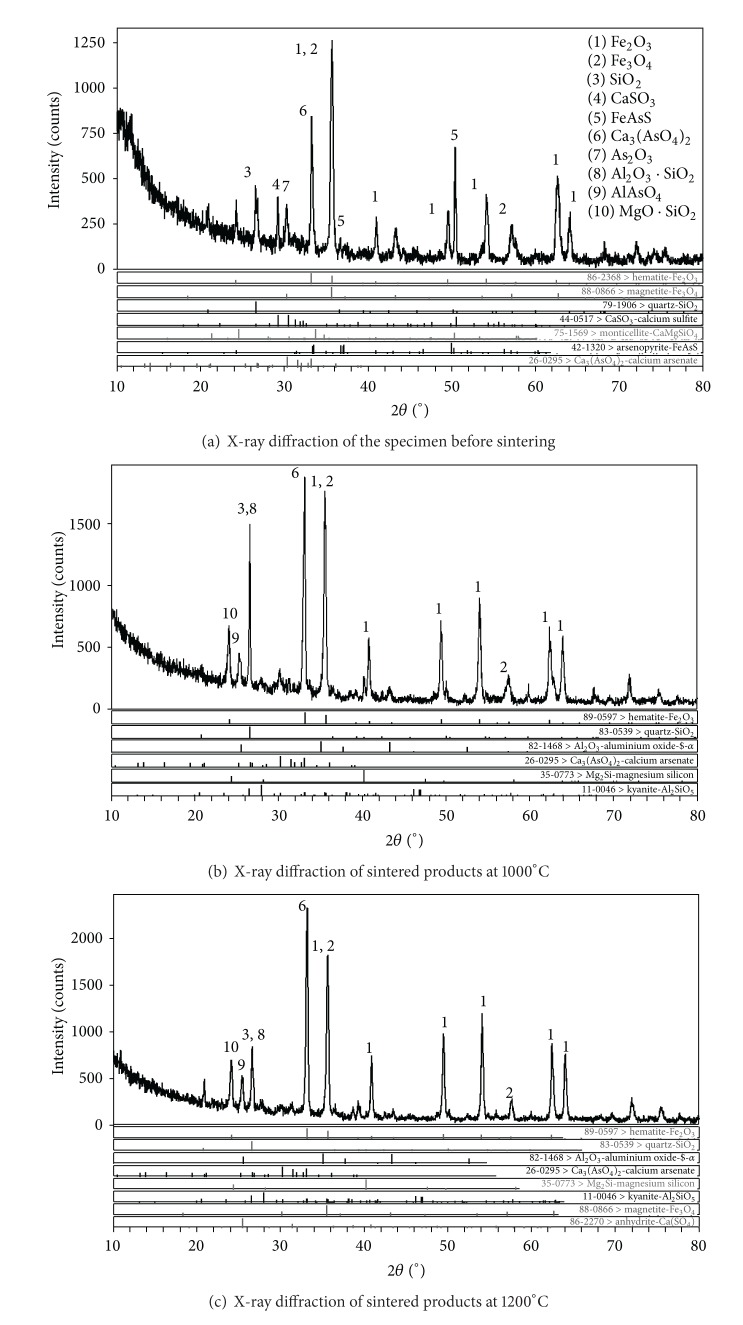
X-ray diffraction of sintered products at different sintering temperatures.

Figure 6 shows the XRD data of sintered products at different sintering temperatures. Hematite (Fe_2_O_3_), calcium sulfite (CaSO_3_), sulfur arsenic iron (FeAsS), quartz (SiO_2_), and arsenic trioxide (As_2_O_3_) are detected originally in the specimen before sintering. Comparing the mineral phases before sintering and after sintering at 1000°C, it was found that the intensities of the peaks associated with FeAsS, CaSO_3_, and As_2_O_3_ decrease as sintering temperature increases, and the peak of AlAsO_4_, Ca_3_(AsO_4_)_2_, MgO·SiO_2_, and Al_2_O_3_·SiO_2_ mineral phases was enhanced. It is suggested that when the sintering temperature increases to 1000°C, several reactions, as follows, take place. When the sintering temperature increased from 1000°C to 1200°C, material structures in the sintered products were not changed significantly:

(1) $$\begin{array}{c} 3\text{FeAsS}+7.25{\text{O}}_{\text{2}}=\text{F}{\text{e}}_{3}{\text{O}}_{\text{4}}+1.5\text{A}{\text{s}}_{\text{2}}{\text{O}}_{\text{3}}+3\text{S}{\text{O}}_{\text{2}} \end{array}$$

(2) $$\begin{array}{c} 3\text{CaO}+\text{A}{\text{s}}_{\text{2}}{\text{O}}_{\text{3}}+{\text{O}}_{2}=\text{C}{\text{a}}_{\text{3}}{\left(\text{As}{\text{O}}_{\text{4}}\right)}_{2} \end{array}$$

(3) $$\begin{array}{c} \text{A}{\text{l}}_{\text{2}}{\text{O}}_{\text{3}}+\text{A}{\text{s}}_{\text{2}}{\text{O}}_{\text{3}}+{\text{O}}_{2}=2\text{AlAs}{\text{O}}_{\text{4}} \end{array}$$

(4) $$\begin{array}{c} \text{F}{\text{e}}_{\text{3}}{\text{O}}_{\text{4}}+1.5\text{A}{\text{s}}_{\text{2}}{\text{O}}_{\text{3}}+1.75{\text{O}}_{\text{2}}=3\text{FeAs}{\text{O}}_{\text{4}} \end{array}$$

(5) $$\begin{array}{c} 2\text{CaS}{\text{O}}_{\text{3}}+{\text{O}}_{2}=2\text{CaS}{\text{O}}_{\text{4}} \end{array}$$

Table 5 shows the results of Gibbs-free energy (Δ*G*) calculation in the heating of FeAsS, −1690 kcal~−2004 kcal, which indicated that the reaction was spontaneously. In this analysis, FeAsS was reacted as (1), As_2_O_3_ was generated, and FeAsS disappeared. As_2_O_3_ can react with Ca, Fe, and Al spontaneously as (2), (3), and (4). When the temperature was lower than 1200°C, the reaction sequence was CaO > Al_2_O_3_ > Fe_3_O_4_, and thus Ca_3_(AsO_4_)_2_ and AlAsO_4_ were generated when it was less than 1200°C, which enhanced the phase peak.

FeAsS is natural mineral, and it is poorly soluble in water. Ca_3_(AsO_4_)_2_ is slightly soluble in water and its solubility is between 0.01 and 12.6 mg/L in water of different pH. arsenic trioxide is soluble in water. Thus, as the sintering temperature increased to 1000°C from room temperature, the leaching concentration of As increased correspondingly.

### 3.5. SEM Microstructure Observation of Sintered Products

**Figure 7:**
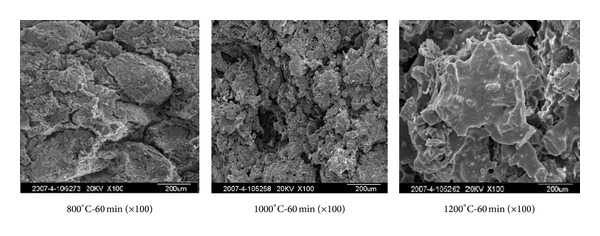
SEM of sintered products at different sintering temperatures.

Figure 7 shows the SEM of sintered products at different sintering temperatures. At 800°C, the sintered body was loosely accumulated as particles; at 1000°C, the specimen began to melt; at 1200°C, the surface of sintered products appeared to be bonded and small amount of liquid was generated. Liquid phase made particles bond with each other; Ca_3_(AsO_4_)_2_ was enveloped in molten state and transformed into chemical bond of Si-Ca arsenate, which greatly reduced the leaching concentration of As.

## 4. Conclusions

1. In the process of sintering As-containing residues at high temperatures, the total amount of As in sintered products changed insignificantly, and the fixed rate of As was higher than 90% at 800~1300°C.
2. Sintering temperature had significant impact on the leaching characteristics of As in sintered products. With HVEP, the higher the sintering temperature, the lower the As leaching concentration. At 1200°C, the leaching concentration of As was 0.002 mg/L. In TCLP, the leaching concentration of As was the lowest at 1200°C, 0.0017 mg/L. Thus from the aspect of environmental security, the best sintering temperature is 1200°C.
3. Sintering time also had significant impact on the leaching characteristics of As in sintered products. With HVEP, the longer the sintering time, the lower the As leaching concentration. From 0 min to 45 min, the concentration was reduced significantly from 0.568 mg/L to 0.003 mg/L. With TCLP, at 30 min, the concentration reduced to 0.0023 mg/L and as the time extended >30 min, the concentration changed a little. Thus the sintering time must be no less than 45 min for the lower leaching concentration of As.
4. In the sintering of As-containing residues, if it is lower than 1000°C, FeAsS will be oxidized and generate As_2_O_3_, which will react with Ca, Al, and Fe to generate corresponding compounds, mainly Ca_3_(AsO_4_)_2_ and AlAsO_4_. FeAsS is poorly soluble in water while Ca_3_(AsO_4_)_2_ is slightly soluble in water. Thus, when it is lower than 1000°C, the leaching concentration of As in sintered products will increase.
5. When the sintering temperature is up to 1200°C, sintered products will be molten; Ca_3_(AsO_4_)_2_ was enveloped in molten state and transformed into chemical bond of Si-Ca arsenate, which greatly reduces the leaching concentration of As.

## Figures and Tables

**Table 1:** Chemical composition of experimental materials.

	Composition of As-containing residues (wt%)	Composition of clay (wt%)	Composition of coal ash (wt%)
SiO_2_	10.10	57.60	46.83
Al_2_O_3_	3.16	14.65	38.86
Fe_2_O_3_	69.78	6.59	4.66
CaO	4.97	11.47	4.65
MgO	1.95	2.32	0.49
K_2_O	0.52	4.23	1.08

**Table 2:** Concentrations and HVEP results of heavy metals in residues.

Heavy metals	Content of trace elements (mg/kg)	HVEP leaching results of heavy metals (mg/L)	Identification Standard for hazardous wastes [9] (mg/L)	Environmental Quality Standards for surface water IV [10] (mg/L)
Cu	128.85	n.d.	100	1.0
Zn	1279.24	n.d.	100	2.0
Cd	n.d.	n.d.	1	0.005
Pb	212.56	n.d.	5	0.05
Cr	n.d.	n.d.	15	0.05
Be	n.d.	n.d.	0.02	—
Ba	1046.80	0.84	100	—
Ni	n.d.	n.d.	5	—
Ag	n.d.	n.d.	5	—
As	5552.40	8.35	5	0.1
Se	n.d.	n.d.	1	0.1

**Table 3:** The As leaching concentration of sintered products at different sintering temperatures.

Sintering temperature (°C)	HVEP (mg/L)	TCLP (mg/L)
0^(1)^	0.568	0.751
1000	0.962	1.444
1050	0.703	1.3
1100	0.315	0.925
1200	0.002	0.0017
1300	0.001	0.0014
1350	0.001	0.0008

^(1)^The specimen was not sintered at 0°C.

**Table 4:** The As leaching concentration of sintered products for different sintering times.

Sintering time (min)	HVEP (mg/L)	TCLP (mg/L)
0^(1)^	0.568	0.751
5	0.482	1.239
10	0.317	0.554
15	0.108	0.194
30	0.086	0.0023
45	0.003	0.0011
60	0.002	0.0017
120	n.d.	0.0009

^(1)^The specimen was not sintered.

**Table 5:** Δ*G* at different sintering temperatures.

*T* (°C)	*G* (kcal)			
	Equation (1)	Equation (2)	Equation (3)	Equation (4)
600	−2004.27	−129.25	−107.29	−71.83
800	−1919.60	−116.56	−101.15	−57.67
1000	−1832.31	−103.80	−94.99	−43.48
1100	−1787.48	−97.42	−91.95	−36.45
1200	−1741.82	−91.05	−88.94	−29.48
1300	−1695.31	−84.70	−85.97	−22.59
